# The effects of exposure to O_2_- and HOCl-nanobubble water on human salivary microbiota

**DOI:** 10.1038/s41598-023-48441-6

**Published:** 2023-11-30

**Authors:** Ken Sagara, Shota Kataoka, Akihiro Yoshida, Toshihiro Ansai

**Affiliations:** 1 Division of Community Oral Health Development, Kyushu Dental University, 2-6-1 Manazuru, Kokurakita-ku, Kitakyushu, 803-8580 Japan; 2 Deparment of Oral Microbiology, Matsumoto Dental University, Shiojiri, Japan

**Keywords:** Microbiology, Molecular biology

## Abstract

Nanobubbles of gas remain dissolved in water for longer periods than ordinary bubbles, and exhibit unique physicochemical and biological properties. As a result, nanobubble water (NBW) is finding widespread use many applications, such as cleaning in the industry and purification of lake water. The ozone NBW (O_3_-NBW), in particular, has been used in clinical dentistry; however, it has several disadvantages, including the instability of ozone, which is spontaneously converted to molecular oxygen (O_3_ to O_2_), and its broad range of antibacterial activity, which can disrupt the oral microbiota. Therefore, the use of NBW in dental medicine requires greater evaluation. Here, we examined the effects of oxygen and hypochlorite NBW (O_2_-NBW and HOCl-NBW, respectively) on the microbiota in human saliva in 16 male patients (35–75 years old; median: 53.5 years) using multiple assays, including next generation sequencing analysis. 16S rRNA gene sequencing revealed no significant changes in both alpha-diversity and beta-diversity. Principal Coordinate Analysis (PCoA) revealed two subclusters in both unweighted and weighted UniFrac distances. Overall, the results revealed that HOCl-NBW exposure of saliva may lead to inhibition or delay in oral biofilm formation while maintaining the balance of the oral microbiome. These results can lead to the development of a novel type of mouthrinse for prevention of oral infectious diseases.

## Introduction

Nanobubbles (NBs) are gaseous bubbles with diameters less than 1 μm, typically in the range of 100 nm^1^. Due to their many favorable properties, the NBs are being increasingly applied in a wide range of fields. Compared with normal water, stably existing NB in water endow the water with properties of a colloid.

The effects nanobubble water (NBW) depend on the gas inside the bubbles. In the environmental field, ozone NB (O_3_-NB) is widely used in floatation and in ozonation processes for the treatment of wastewater^2,3^. In agriculture and aquaculture, oxygen NBW (O_2_-NBW) accelerates the growth of oxygen-requiring aquatic organisms such as plants and fish^4–6^. In the food industry, NB is used for sterilization of the food by treatment with CO_2_-NB or O_3_-NB, thus maintaining both safety and hygiene^7,8^.

The NBW that have been applied in the field of dentistry are mainly O_3_-NBW. For example, Hayakumo et al.^9^ performed mechanical subgingival debridement using O_3_-NBW in periodontal disease patients and found that the total number of bacteria in the subgingival plaque was significantly reduced compared to the control group, also resulting in clinical improvement including reduction of the probing depth (PD).

While reports of the use of NBW in dental medicine and its effects on oral microbiota are relatively scarce, NBW studies in the gut microbiota have recently been published. Specifically, Guo et al.^10^ examined the effects of N_2_-NBW and H_2_-NBW on murine gut microbiota in mice, and found that N_2_-NBW supplementation increased the number of beneficial genera such as *Clostridium* and *Caprococcus*, while H_2_-NBW decreased the number of pathogenic genera such as *Mucispirillum* and *Helicobacter*^10^. Thus, use of NBW may help to optimize the composition of gut microbiota. In the environmental field, adding O_2_-NB modified minerals induced changes in the microbial community structure in surface sediments and strengthened the role of nitrobacteria, denitrifying bacteria, and ammonia oxidation bacteria^11^. However, to date there are no reports on how NBW affect the oral microbiota.

Hypochlorous acid (HOCl) water has been traditionally used for bacterial disinfection in the food industry^12^. HOCl is the acidic equilibrium chemical variant of hypochlorite (OCl^-^) that exists as the predominant species in the pH range 6.6–6.8. Hakim et al.^13^ reported that sprayed HOCl-water was able to inactivate *E. coli* and *Salmonella*, and prevent disease transmission. To our knowledge, however, no studies on HOCl-NBW, including the use of HOCl-NBW in the oral cavity, have been reported.

As an example, the use O_2_-NB, Wang et al. developed a material containing O_2_-NB and reported that it was able to significantly increase dissolved oxygen and oxidation reduction potential in anaerobic systems^14^. Thus, the use of HOCl-NBW with their inactivating effect on bacteria, and O_2_-NBW with their purifying effect on anaerobic environments, may be useful in maintaining a favorable oral environment.

An increasing number of recent studies have focused on the “ecological hypothesis” that the oral environment alters the dental plaque microbiota, leading to the pathogenesis of dental caries and periodontal disease^15^. For example, periodontal disease is caused by dysbiosis of subgingival microbial communities^16^. Also, dental caries is considered to be caused by acids produced by the overall dental plaque microbiota rather than by specific pathogens^17^.

We therefore hypothesized that O_2_-NBW and HOCl-NBW could affect the composition of oral microbiota and the relative abundance and prevalence of main periodontitis-associated taxa. The purpose of this study was to examine the effects of O_2_-NBW and HOCl-NBW on the composition of oral microbiota via analyses of salivary microbiota.

## Results

### Clinical parameters of the participants

Table 1 shows clinical parameter of the participants. The median age was 53.5 years (Interquartile range (IQR) 45.8–68.0), and the median number of teeth was 25.5 (IQR 23.0–28.0). Of the total number of 153 periodontal sites, the median number of pockets less than 3 mm was 123.50 (IQR 101.80–147.00), the median number of pockets 4 mm was 16 (IQR 3.75–28.25), and the median number of pockets greater than 5 mm was 1.5 (IQR 0.00–8.00).

**Table 1:** Clinical characteristic of the participants (N = 16).

Age	53.50	[45.75–68.00]
N of teeth	25.50	[22.75–28.00]
BOP	1.45	[0.00–46.54]
N of periodontal sites	153.00	[136.50–168.00]
N of PD ≤ 3 mm	123.50	[101.80–147.00]
N of PD = 4 mm	16.00	[3.75–28.25]
N of PD ≥ 5 mm	1.50	[0.00–8.00]

Numbers in parentheses indicate interquartile range (IQR). Measurement of periodontal pocket was done by probing six sites per tooth. Statistical analysis was performed with Mann–Whitney U test between groups. Data indicate median.

*BOP* bleeding on probing, *PD* pocket depth, *N* number.

#### Alpha- and beta-diversity analyses

**Figure 1:**
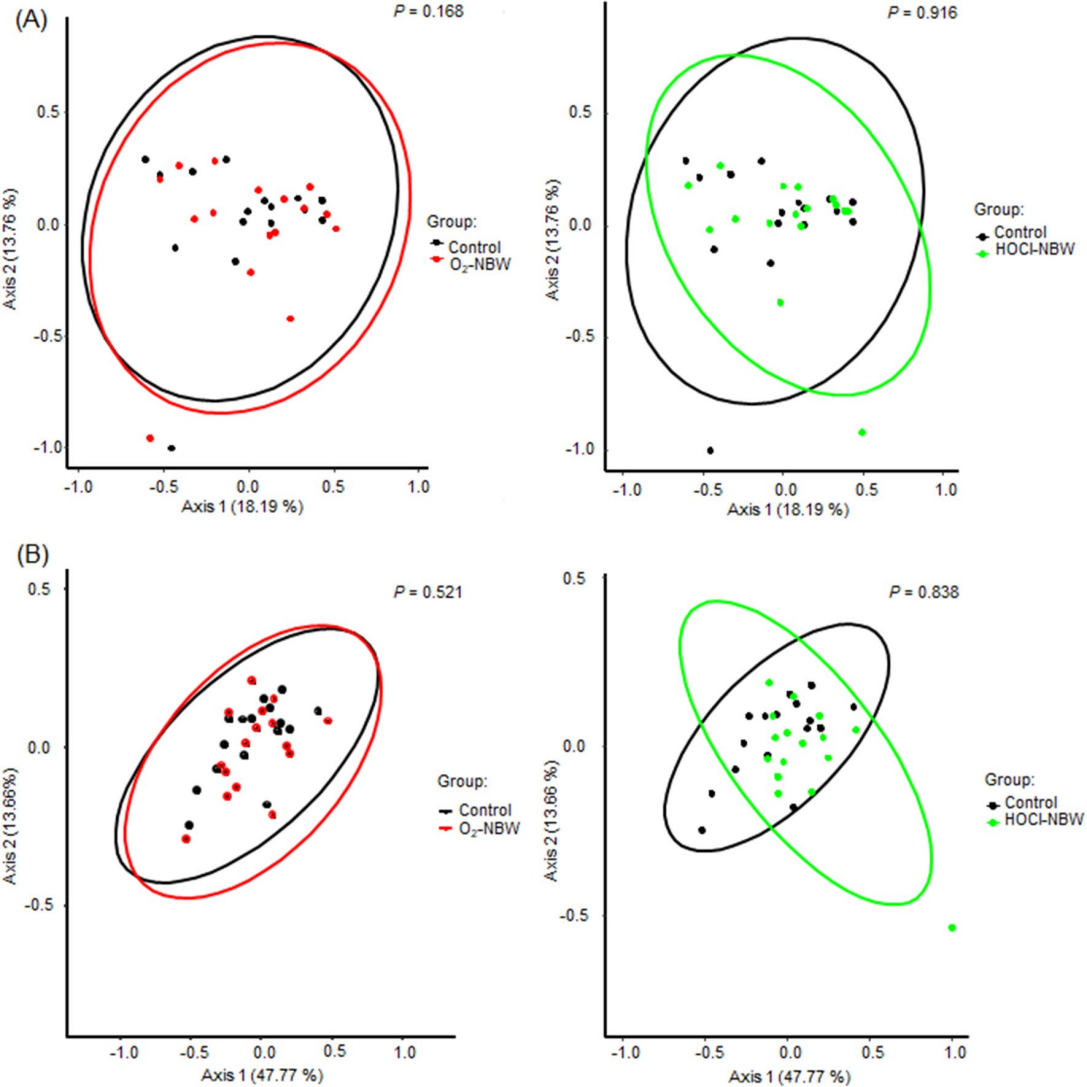
Beta-diversity of unweighted UniFraq distance (**A**) and weighted UniFraq distance (**B**). Colored dots indicate individual sample groups: Black: Control; red: O_2_-NBW; green: HOCl-NBW. Colored circles indicate groups exposed to NBW; Black: Control; Red: O_2_-NBW; Green: HOCl-NBW.

In this study, salivary microbiota composition of 16 patients was studied based on the sequencing of the 16S rRNA gene. The samples provided 2,092,625 quality reads corresponding to the V3–V4 regions of the 16S rRNA gene sequences, which were subsequently assigned to 308 species-level operational taxonomic units (OTUs) based on ~ 97% sequence similarity. We investigated the changes of alpha-diversity due to exposure to NBW. In observed features, O_2_-NBW and HOCl-NBW tended to decrease alpha-diversity relative to the control; however, the differences were not significant (*P* = 0.85). Shannon index also did not show significant differences (*P* = 0.79) (Supplementary Fig. 1). Figure 1 shows the scatter diagram of beta-diversity based on Principal Coordinate Analysis (PCoA). In the Unweighted UniFraq distance, there was no significant difference between control and O_2_-NBW (*P* = 0.168) or between control and HOCl-NBW (*P* = 0.916) (Fig. 1A). Similarly, there was no significant difference between control and O_2_-NBW or HOCl-NBW at the Weighted UniFrac distance (Fig. 1B, P = 0.521; *P* = 0.828, respectively).

#### Effects on salivary microbiota with NBW

Supplementary Fig. 2 shows the relative frequencies of the different salivary bacteria. The bacterial genera, based on detection in 1% or more of the total population of the salivary microbiome, were composed of 71 OTUs (frequency > 0.001) (Supplementary Fig. 2A). Specifically, 14 major genera including *Prevotella*, *Streptococcus*, *Veillonella*, *Neisseria*, *Haemophilus*, *Leptotrichia*, *Porphyromonas*, *Fusobacterium*, *Rothia*, *Graulicatella*, *Alloprevotella*, *Campylobacter*, *Atopobium*, *Saccharibacteria* (TM7) [G-1] were detected. In similar analyses, bacterial species that were detected in 1% and more of the salivary microbiome, constituted 166 OTUs (frequence > 0.001) and included 25 major species, namely *Prevotella melaninogenica, Haemophilus parainfluenzae, Streptococcus salivarius, Neisseria* spp*., Porphyromonas pasteri, Veillonella dispar, Streptococcus* spp*., Rothia mucilaginosa, Fusobacterium periodonticum, Veillonella atypica, Leptotrichia* sp. HMT417*, Prevotella pallens, Veillonella parvula, Veillonella rogosae, Prevotella* spp., *Granulicatella adiacens, Leptotrichia* sp. HMT221*, Streptococcus parasanguinis* clade411*, Neisseria subflava, Prevotella* sp. HMT313*, Prevotella salivae, Campylobacter concisus, Leptotrichia* sp. HMT215, *Saccharibacteria* (TM7) [G-1] *bacterium* HMT352*, Atopobium parvulum.*

### Comparison of abundance in bacterial genera and species NBW-exposed saliva

We next investigated the relative abundance in the control and exposed groups by bacterial genera. Repeat measures ANOVA for the 14 bacterial genera with detection rates greater than 1% showed that only the genus *Porphyromonas* had a significant association among the three groups. Multiple testing also revealed significant associations between control and O_2_-NBW (*P* = 0.044) and between control and HOCl-NBW (*P* = 0.007) in the genus *Porphyromonas* (Table 2). Also, we investigated the relative abundance in the control and exposed groups by bacterial species. Repeated measures analysis of variance for the 25 bacterial species with detection rates greater than 1% showed that only *P. pasteri* was significantly associated among the three groups (*P* = 0.008). Multiple testing also showed a significant reduction (1.066%) in *P. pasteri* (*P* = 0.028) between control and HOCl-NBW (Table 3).

**Table 2:** Comparison of abundance in bacterial genera following exposure to NBW.

	Control	O_2_-NBW	HOCl-NBW	O_2_-NBW	HOCl-NBW
*Prevotella*	20.227	19.808	17.411	–	–
*Streptococcus*	13.800	14.496	22.407	–	–
*Veillonella*	13.395	12.857	12.958	–	–
*Neisseria*	8.947	9.404	7.874	–	–
*Haemophilus*	8.210	6.688	8.599	–	–
*Leptotrichia*	7.187	7.245	4.593	–	–
*Porphyromonas*	6.770	5.960	5.514	*	*
*Fusobacterium*	3.945	3.930	3.977	–	–
*Rothia*	3.760	5.429	3.294	–	–
*Granulicatella*	1.798	1.596	2.055	–	–
*Alloprevotella*	1.400	1.083	1.179	–	–
*Campylobacter*	1.234	1.433	0.961	–	–
*Atopobium*	1.189	0.934	0.698	–	–
*Saccharibacteria* (TM7) [G-1]	1.120	0.946	1.123	–	–

Relative abundance of oral bacterial genera in NBW-exposed samples. Bacterial genera of relative abundance ≥ 1% are shown. Data indicate median.

*P < 0.05.

− : NS (Not Significant). Statistical analysis was performed with repeated-measures ANOVA between control and O_2_-NBW or HOCl-NBW.

**Table 3:** Comparison of abundance in bacterial species following exposure to NBW.

	Control	O_2_-NBW	HOCl-NBW	O_2_-NBW	HOCl-NBW
*Prevotella melaninogenica*	9.660	8.674	9.317	–	–
*Haemophilus parainfluenzae*	8.011	6.504	8.540	–	–
*Streptococcus salivarius*	7.725	8.052	10.223	–	–
*Neisseria* spp.	7.447	7.859	6.474	–	–
*Porphyromonas pasteri*	6.000	5.282	4.934	–	*
*Veillonella dispar*	5.305	5.094	5.101	–	–
*Streptococcus* spp*.*	4.715	5.096	9.796	–	–
*Rothia mucilaginosa*	3.679	5.326	3.294	–	–
*Fusobacterium periodonticum*	3.594	3.710	3.708	–	–
*Veillonella atypica*	3.418	3.385	2.892	–	–
*Leptotrichia* sp. HMT417	2.569	2.399	1.981	–	–
*Prevotella pallens*	2.428	2.444	2.284	–	–
*Veillonella parvula*	2.366	2.169	2.507	–	–
*Veillonella rogosae*	2.223	1.982	2.201	–	–
*Prevotella* spp.	2.140	2.418	1.759	–	–
*Granulicatella adiacens*	1.737	1.531	2.001	–	–
*Leptotrichia* sp. HMT221	1.484	1.253	0.938	–	–
*Streptococcus parasanguinis* clade411	1.360	1.348	2.320	–	–
*Neisseria subflava*	1.350	1.384	1.286	–	–
*Prevotella* sp. HMT313	1.320	1.189	1.022	–	–
*Prevotella salivae*	1.226	1.283	0.946	–	–
*Campylobacter concisus*	1.182	1.433	0.961	–	–
*Leptotrichia* sp. HMT215	1.116	1.590	1.020	–	–
*Saccharibacteria* (TM7) [G-1] *bacterium* HMT352	1.105	0.946	1.123	–	–
*Atopobium parvulum*	1.079	0.854	0.698	–	–

Relative abundance of oral bacterial species in NBW-exposed samples. Bacterial species of relative abundance ≥ 1% are shown. Data indicate median.

*P < 0.05.

− : NS (Not Significant). Statistical analysis was performed with repeated-measures ANOVA between control and O_2_-NBW or HOCl-NBW.

### Cluster analysis based on PCoA

**Figure 2:**
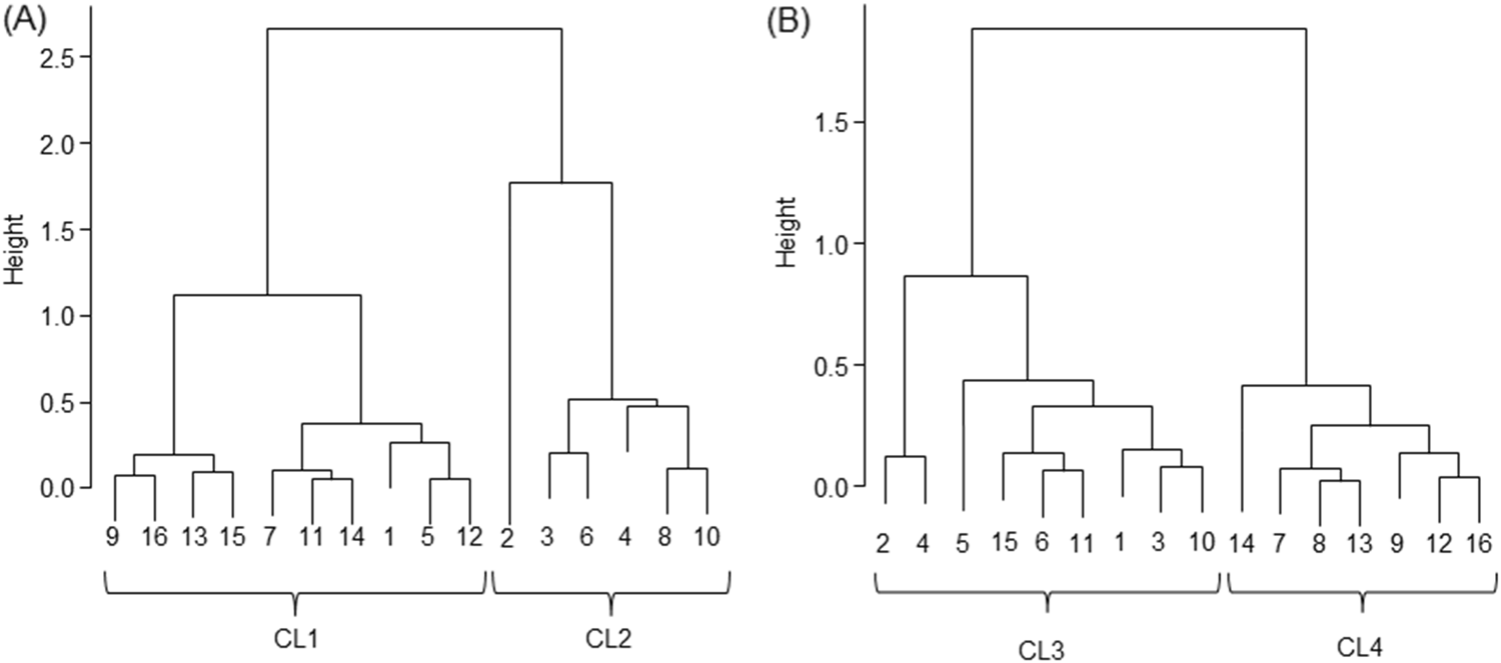
Results of cluster analysis of relative abundance in oral microbiome (N = 16). (**A**) Unweighted cluster. (**B**) Weighted cluster. Stratified cluster analyses were performed according to the Ward method based on the results of PCoA. Numbers indicate sample ID. Clustering was performed using the Ward method with Euclidian Distance.

Figure 2 shows the results of the hierarchical cluster analysis by Ward’s method based on the results of the PCoA, which revealed two subclusters in terms of both Unweighted UniFraq distance (Fig. 2A) and Weighted UniFraq distance (Fig. 2B). In Fig. 2A, CL1 and CL2 were formed, with CL1 having 10 subjects and CL2 having 6 subjects. In Fig. 2B, CL3 and CL4 were formed, with CL3 comprising 9 subjects and CL4 7 subjects.

#### Comparison between two cluster with different susceptibility to expose to NBW

Supplementary Fig. 3 shows the results of the principal coordinates analysis of the Unweighted UniFraq distance (A, B), and the Weighted UniFrac distance (C, D). Supplementary Fig. 3A shows the results between Control and O_2_-NBW, and Supplementary Fig. 3B shows the results between Control and HOCl-NBW. In Supplementary Fig. 3A, there was no significant difference between the two groups in CL1 (P = 0.536), while in CL2 there was a significant difference between the two groups (P = 0.033). On the other hand, in Supplementary Fig. 3B, there was no significant difference between CL1 and CL2.

In contrast, Supplementary Fig. 3C,D show the results of the principal coordinates analysis of the Weighted UniFraq distance. Supplementary Fig. 3C shows the results for control and O_2_-NBW, and Supplementary Fig. 3D shows the results for control and HOCl-NBW. There were no significant differences between the two groups for both CL3 and CL4 in Supplementary Fig. 3C,D.

### Relative abundance of bacterial genera and species

We investigated the relative abundance of bacterial genera in CL1 and CL2 in the Unweighted cluster; no bacterial genera were significantly different in both CL1 and CL2. Also, in bacterial species, no bacterial species were found to have a significant difference between CL1 and CL2 (Supplementary Table 1).

On the other hand, in the relative abundance of bacterial genera in CL3 in the weighted clusters, the only significant reduction (1.186%) between Control and HOCl-NBW was observed in the genus *Porphyromonas* (Table 4). However, no bacterial genus showed significant differences in CL4. In the relative abundance of bacterial species, only *P. pasteri* showed significant reduction (0.921%) among the bacterial species in the CL3. On the other hand, no significant differences were found among the bacterial species in the CL4 (Table 5).

**Table 4:** Relative abundance of bacterial genera in weighted UniFraq: CL3 and CL4.

	Control	O_2_-NBW	HOCl-NBW	O_2_-NBW	HOCl-NBW
Genera: CL3					
*Prevotella*	24.248	23.196	18.882	–	–
*Streptococcus*	8.442	9.993	23.029	–	–
*Veillonella*	15.965	14.485	14.542	–	–
*Neisseria*	6.79	7.065	5.833	–	–
*Haemophilus*	6.003	4.056	6.434	–	–
*Leptotrichia*	10.827	10.97	6.401	–	–
*Porphyromonas*	4.926	4.152	3.74	–	*
*Fusobacterium*	4.032	3.643	3.694	–	–
*Rothia*	3.509	5.613	2.741	–	–
*Granulicatella*	0.752	0.51	1.687	–	–
*Alloprevotella*	1.714	1.402	1.495	–	–
*Campylobacter*	1.631	2.011	1.106	–	–
*Atopobium*	1.917	1.392	1.008	–	–
*Saccharibacteria* (TM7) [G-1]	1.408	1.096	1.229	–	–
Genera: CL4					
*Prevotella*	15.056	15.452	15.519	–	–
*Streptococcus*	20.689	20.286	21.609	–	–
*Veillonella*	10.092	10.764	10.921	–	–
*Neisseria*	11.721	12.41	10.498	–	–
*Haemophilus*	11.048	10.072	11.383	–	–
*Leptotrichia*	2.508	2.455	2.268	–	–
*Porphyromonas*	9.142	8.285	7.794	–	–
*Fusobacterium*	3.833	4.299	4.341	–	–
*Rothia*	4.084	5.193	4.004	–	–
*Granulicatella*	3.142	2.993	2.528	–	–
*Alloprevotella*	0.996	0.673	0.773	–	–
*Campylobacter*	0.724	0.691	0.775	–	–
*Atopobium*	0.254	0.346	0.298	–	–
*Saccharibacteria* (TM7) [G-1]	0.751	0.753	0.987	–	–

Data indicate median.

*P < 0.05.

− : NS. Statistical analysis was performed with repeated-measures ANOVA.

**Table 5:** Relative abundance of bacterial species in weighted UniFraq: CL3 and CL4.

	Control	O_2_-NBW	HOCl-NBW	O_2_-NBW	HOCl-NBW
Species: CL3					
*Prevotella melaninogenica*	9.559	7.872	8.616	–	–
*Haemophilus parainfluenzae*	5.822	3.894	6.329	–	–
*Streptococcus salivarius*	5.615	6.768	10.208	–	–
*Neisseria* spp.	6.488	6.773	5.664	–	–
*Porphyromonas pasteri*	3.987	3.307	3.066	–	*
*Veillonella dispar*	6.581	6.166	6.202	–	–
*Streptococcus* spp.	1.995	2.440	10.061	–	–
*Rothia mucilaginosa*	3.416	5.429	2.741	–	–
*Fusobacterium periodonticum*	3.705	3.566	3.539	–	–
*Veillonella atypica*	5.299	5.132	4.217	–	–
*Leptotrichia* sp. HMT417	3.889	3.881	2.996	–	–
*Prevotella pallens*	2.931	2.978	2.767	–	–
*Veillonella parvula*	2.535	1.952	2.476	–	–
*Veillonella rogosae*	1.400	0.830	1.295	–	–
*Prevotella* spp.	2.829	3.359	2.119	–	–
*Granulicatella adiacens*	0.644	0.394	1.590	–	–
*Leptotrichia* sp. HMT221	2.354	2.041	1.529	–	–
*Streptococcus parasanguinis* clade411	0.832	0.785	2.638	–	–
*Neisseria subflava*	0.058	0.071	0.063	–	–
*Prevotella* sp. HMT313	2.184	1.718	1.449	–	–
*Prevotella salivae*	1.829	1.899	1.285	–	–
*Campylobacter concisus*	1.548	2.011	1.106	–	–
*Leptotrichia* sp. HMT215	1.439	2.069	1.187	–	–
*Saccharibacteria* (TM7) [G-1] *bacterium* HMT352	1.380	1.096	1.229	–	–
*Atopobium parvulum*	1.722	1.249	1.008	–	–
Species: CL4					
*Prevotella melaninogenica*	9.789	9.705	10.219	–	–
*Haemophilus parainfluenzae*	10.824	9.858	11.383	–	–
*Streptococcus salivarius*	10.437	9.703	10.243	–	–
*Neisseria* spp.	8.680	9.256	7.515	–	–
*Porphyromonas pasteri*	8.587	7.822	7.337	–	–
*Veillonella dispar*	3.664	3.715	3.687	–	–
*Streptococcus* spp.	8.213	8.512	9.455	–	–
*Rothia mucilaginosa*	4.017	5.193	4.004	–	–
*Fusobacterium periodonticum*	3.451	3.894	3.924	–	–
*Veillonella atypica*	0.999	1.139	1.188	–	–
*Leptotrichia* sp. HMT417	0.872	0.494	0.676	–	–
*Prevotella pallens*	1.782	1.756	1.662	–	–
*Veillonella parvula*	2.148	2.448	2.546	–	–
*Veillonella rogosae*	3.280	3.462	3.367	–	–
*Prevotella* spp.	1.256	1.209	1.296	–	–
*Granulicatella adiacens*	3.142	2.993	2.528	–	–
*Leptotrichia* sp. HMT221	0.366	0.241	0.178	–	–
*Streptococcus parasanguinis* clade411	2.039	2.071	1.911	–	–
*Neisseria subflava*	3.012	3.073	2.858	–	–
*Prevotella* sp. HMT313	0.209	0.509	0.474	–	–
*Prevotella salivae*	0.451	0.491	0.510	–	–
*Campylobacter concisus*	0.711	0.691	0.775	–	–
*Leptotrichia* sp. HMT215	0.701	0.974	0.806	–	–
*Saccharibacteria* (TM7) [G-1] *bacterium* HMT352	0.751	0.753	0.987	–	–
*Atopobium parvulum*	0.254	0.346	0.298	–	–

Data indicate median.

*P < 0.05.

–: NS. Statistical analysis was performed with repeated-measures ANOVA.

### Clinical parameters of the participants according to cluster

Tables 6 and 7 show the clinical parameters of the subjects according to cluster. Table 6 shows the Unweighted results; the categories that showed significant differences between CL1 and CL2 were the number of probing pocket depth (PD)s less than 3 mm, the number of PDs 4 mm, and the number of PDs greater than 5 mm. No significant differences were found in the other categories. Table 7 shows the weighted results, where the category that showed a significant difference between CL3 and CL4 was the number of PDs of 4 mm. No significant differences were found in the other categories.

**Table 6:** Clinical characteristics of the subjects according to cluster: unweighted.

Unweighted	CL1 (N = 10)		CL2 (N = 6)		*P*-value
	Median	(IQR)	Median	(IQR)	
Age	53.50	[47.50–61.00]	58.50	[46.00–68.00]	1.000
N of teeth	24.50	[23.25–26.50]	24.50	[23.25–26.50]	0.477
BOP	0.00	[0.00–8.65]	34.72	[8.97–53.00]	0.101
N of periodontal sites	147.00	[139.50–159.00]	162.00	[135.00–168.00]	0.477
N of PD ≤ 3 mm	144.00	[126.00–153.00]	110.00	[95.25–115.75]	0.034
N of PD = 4 mm	5.50	[3.00–15.00]	29.50	[20.25–39.50]	0.023
N of PD ≥ 5 mm	0.00	[0.00–1.75]	8.50	[8.00–10.50]	0.004

Data indicate median.

Statistical analysis was performed with the Mann–Whitney U test.

*BOP* bleeding on probing, *PD* pocket depth, *IQR* interquartile range.

**Table 7:** Clinical characteristics of the subjects according to cluster: weighted.

Weighted	CL3 (N = 9)		CL4 (N = 7)		*P*-value
	Median	(IQR)	Median	(IQR)	
Age	62.00	[52.00–68.00]	49.00	[45.00–55.50]	0.314
N of teeth	24.00	[22.00–28.00]	26.00	[24.50–28.00]	0.557
BOP	5.07	[0.00–48.77]	0.00	[0.00–36.23]	0.658
N of periodontal sites	144.00	[132.00–168.00]	156.00	[147.00–168.00]	0.557
N of PD ≤ 3 mm	115.00	[92.00–129.00]	141.00	[123.50–156.00]	0.153
N of PD = 4 mm	27.00	[17.00–32.00]	4.00	[2.50–11.50]	0.049
N of PD ≥ 5 mm	6.00	[0.00–8.00]	1.00	[0.00–3.00]	0.383

Data indicate median.

Statistical analysis was performed with the Mann–Whitney U test.

*BOP* bleeding on probing, *PD* pocket depth, *IQR* interquartile range.

### Association between PD counts and the effect of HOCl-NBW exposure on *P. pasteri*

**Figure 3:**
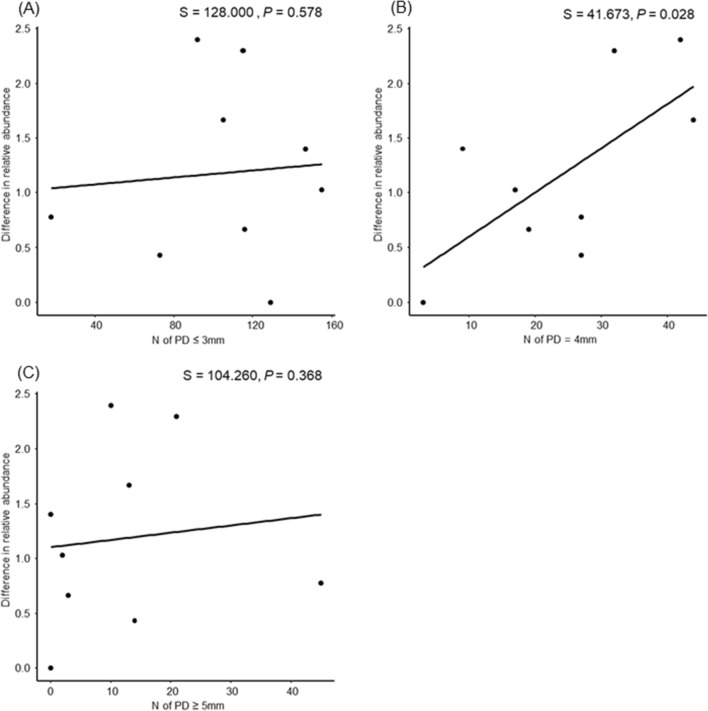
Scatter plots and correlation coefficient tests in CL3 group (N = 9). Spearman’s rank correlation coefficient. Alternative hypothesis: true ρ is greater than 0. The significance level was set at alpha = 0.05. (**A**) Spearman’s rank correlation coefficient − 0.0667 (P = 0.58). (**B**) Spearman’s rank correlation coefficient 0.653 (P = 0.028). (**C**) Spearman’s rank correlation coefficient 0.131(P = 0.37).

Figure 3 shows a scatter plot between PD values and difference in relative abundance in CL3 (N = 9), the cluster where a significant association between Control and HOCl-NBW was observed in Tables 4 and 5. As shown in Fig. 3B, t = 2.45 at PD = 4 mm, indicating that the higher the number of PD = 4 mm, the higher the effect of HOCl-NBW exposure on *P. pasteri.* On the other hand, no significant association was found for PD = 3 mm or less and PD = 5 mm or more. These results suggest that relative abundance of *P. pasteri* is associated with clinical signs of early stage of periodontitis.

## Discussion

In this study, we examined the effects of exposure to two types of NBW, namely O_2_-NBW and HOCl-NBW, and control deionized water (DW) on salivary microbiota. The results showed that: (i) neither alpha-diversity nor beta-diversity was changed by exposure to both NBW; (ii) *Porphyromonas* was the only bacterial genera that showed a significant difference between the control and HOCl-NBW-exposed groups; (iii) Among *Porphyromonas*, only *P. pasteri* was significantly reduced by HOCl-NBW exposure.

This is the first study to examine the relationship between NBW exposure and oral microbiota. To our knowledge there have been no studies examining human microbiota (including the oral microbiota), although there was a study in mice regarding those relationship^10^. According to Guo’s study, no difference in the alpha-diversity was observed, while the beta-diversity between N_2_-NBW and the other two groups (H_2_-NBW and the control) was observed, which means alteration of the species diversity of gut microbiota due to NBW exposure.

On the other hand, HOCl-NBW had no effect on salivary microbiota in this study. It is possible that the exposure of HOCl-NBW seemed to act mildly without significantly disrupting the balance of microbiota. Interestingly, similar findings have been found in ophthalmologic studies: Yang et al.^18^ report that when HOCl is used for eyelid cleaning, there was no significant difference in alpha-diversity before and after eyelid cleaning. Furthermore, it was the same at the phylum level^18^. At present, the mechanism of the effects remains known, as discussed by Yang et al.^18^, but HOCl could conceivably affect the relative abundance of commensal pathogenic bacteria via its broad-spectrum antibacterial effects.

In general, HOCl is highly active against bacterial, viral, and fungal microorganisms, and is active against biofilm^19^. In addition to the stabilization of HOCl by nanobubbling, the increase in water mobility may also contribute to its enhanced antimicrobial effect^20^. Considering the finding that water mobility might influence the composition of gut microbiota^10^, it is quite possible that the same may be true for the inhibitory activity of HOCl-NBW on the oral microbiota.

On the other hand, the effect of O_2_-NBW exposure on salivary microbiota in our study was not clear. The report by Yamaguchi et al.^21^ may be helpful in this regard. This group investigated the effects of CO_2_-NBW, O_2_-NBW, and N_2_-NBW exposure on *E. coli* growth, and found that CO_2_-NBW had a bactericidal effect on *E. coli,* whereas N_2_-NBW and O_2_-NBW did not have a significant bactericidal effect.

In this study, we have discovered a suppressive effective of HOCl-NBW exposure on the growth of *P. pasteri*. Of the newly discovered non-pigmented species of the *Porphyromonas* genus*, P. pasteri* is an anaerobic, weakly saccharolytic, Gram-negative rod, isolated by Sakamoto et al.^22^. According to Guilloux et al*.*^23^, *P. pasteri/P. catoniae* is a member of the healthy oral microbiome, while *P. gingivalis* is a member of the core microbiome in periodontitis. Diao et al*.*^24^ mentioned that the degree of dysbiosis shows a gradual transition of the entire microbial community from healthy to diseased periodontitis. In comparing the relative abundance and prevalence of oral taxa, Lenartova et al*.*^25^ found that the most abundant and prevalent taxa in healthy dental clusters are *S. mitis, S. gordonii,* and *N. flava*, while the relative abundance of red complexes such as *P. gingivalis, T. forsythia,* and *T. denticola* do not reach high values in healthy periodontal and transient areas. The taxa showing a higher prevalence in the transient area were *F. nucleatum* and *P. pasteri.* Considering that the prevalence of *P. pasteri* increases from healthy to transient status, it may serve as a marker of the transient state proceeding to periodontitis. Lenartova^25^, thus offering an avenue to reduce the risk of periodontal disease progression.

At this time, we do not know why *P. pasteri* displays an apparently higher susceptibility to HOCl-NBW exposure. HOCl is a weak acid that tends to dissociate to generate the hypochlorite ion (ClO^−^). The high reactivity of the hypochlorite ion endows it with the ability to form adducts with a large variety of essential biochemicals, such as DNA, RNA, proteins and lipids^26^. Thus, it is plausible that some of these molecules in *P. pasteri* are more easily accessible to hypochlorite due to their unique structural features or location. This query can lead to interesting research for the future.

Limitations of this study include the following: (1) The recruited patients had relatively mild periodontal symptoms (average PD of 3 to 5 mm), and thus, the effect of NBW on severe periodontal disease was not tested. (2) The study design was cross-sectional, so we were unable to examine changes before and after exposure to NBW, as well as continuous changes through multiple exposures. (3) The in vitro anaerobic culture using BHI may not fully replicate the in vivo environment. Specifically, they may differ with respect to the percentage of aerobic or obligatory anaerobic bacteria present. (4) In the present study, the identification of *P. pasteri* was based on ~ 97% similarity of an amplicon derived from 16S RNA. Ideally, however, the most reliable identification should be based on full genome sequencing, which will require either shotgun metagenomics or sequencing of the isolated target species. (5) Generalizability could be considered limited due to small sample size and only male patients. Clinical studies including women are planned for the future. (6) We did not perform safety confirmation experiments, such as effects on cell growth in vivo. However, according to a report from Ono et al*.*^27^, when weak acidic hypochlorous solution was used for drinking by chickens, there were no problems with growth rate at an effective chlorine concentration of 50 ppm and pH of 5.5 to 6.5. In light of this, it seems unlikely that adverse events would occur immediately at the concentrations of NBW used in this study. Further study with a larger sample size and control groups would be necessary to make a more significant conclusion, translatable to population-scale dental health.

The existence of *P. pasteri* in the oral microbiome may offer important clinical considerations and therapeutic interventions in dental medicine. It has been reported, for example, that *P. pasteri* is suspected to act as a bridge organism that coaggregates early and late colonizers, similar to *Fusobacterium nucleatum*^28^. Therefore, controlling the growth of *P. pasteri* using HOCl-NBW exposure may lead to inhibition or delay in oral biofilm formation that causes oral diseases such as dental caries and periodontal disease, while keeping the balance of the oral microbiome. Our result may lead to the development of novel type of clinical applications including mouthrinse for prevention of oral infectious diseases.

## Conclusion

Exposure of the salivary microbiota to HOCl-NBW would make beneficial effects on the oral environment of the host: the exposure did not disrupt the balance of oral microbiota; the exposure may lead to inhibition or delay in oral biofilm formation. Future development of new type of mouthrinse for the prevention of biofilm formation during initial stage of periodontal disease would be expected.

## Methods

### Ethics approval

The studies involving human participants were reviewed and approved by the Institutional Review Board, Kyushu Dental University, Japan (No. 21-17). All patients understood the nature of the study and provided written informed consent. Collected data were managed by ID numbers; personal information, including DNA information, was handled in accordance with the guidelines of the Personal Information Protection Act in Japan.

### Recruitment of study participants

A cohort of 16 patients was recruited from two cooperating private dental offices during the period from October to December, 2021. Inclusion criteria were set for 35–75 years old patients at baseline who had not received any dental treatment including for periodontal disease in the preceding year. Since the existence of gender differences in the pathogenesis of periodontal disease has been reported^29,30^, the subjects in this study were focused on gender males. Exclusion criteria were set as follows; (1) presence of acute periodontal disease, (2) continuous prescription of antibiotics within the past month, (3) local drug delivery systems (LDDS) in the treatment of periodontitis within the past three months, (4) Uncontrolled diabetes (Diabetes was defined as HbA1c > 7%), (5) Steroid therapy for autoimmune diseases, (6) dry mouth with difficulty in saliva sampling. All patients agreed to the purpose of this study with prior written consent.

### Oral examination

All participants were examined by experienced dentists or dental hygienists in two cooperating private dental offices. The clinical items included PD, bleeding on probing (BOP), and the number of teeth present. PD and BOP were conducted from six sites per tooth, that is, three sites from the labial/buccal aspects and the other three sites from the palatal/lingual aspects as mesial, medium, and distal sites for both jaws.

### Saliva sample collection

In this study, saliva was used as a sample to evaluate the salivary microbial community. Saliva samples were collected using the spitting method by well-trained dentists or dental hygienists on the first visit. Participants were asked to chew paraffin gum for 5 min and saliva was put into the tube. Participants refrained from oral cleaning with toothpaste or mouthwash, eating, drinking, and smoking for one hour before beginning collection of the saliva samples. Saliva samples were immediately stored in the ultra-low temperature (− 80 °C) freezer until further processing.

### Generation of NBW

The NBW used in this study was produced by a nanobubble generator (EnH Co., Ltd., Chungnam, Korea); the O_2_-NBW concentration was approximately 40 ppm and the pH was ~ 7.4. The concentration of HOCl-NBW was ~ 50 ppm and pH ~ 5.0. The number of both NBWs produced was about 100 million. pH was measured using LAQUAtwin (AS-pH-22, Horiba Advanced Techno Co., Ltd., Japan) and the number of nanobubbles was measured using NanoSight NS300 (Malvern Panalytical Ltd., UK).

### Exposure to NBW and preparation of saliva sample

The saliva samples obtained from 16 subjects were divided into three groups of equal amounts: control, O_2_-NBW, and HOCl-NBW, as shown in Supplementary Fig. 4. NBW (3.5 ml)—or deionized water (DW) as control—was mixed with 4× concentration of BHI liquid medium. Saliva sample was added into the medium, and anaerobically incubated at 37 °C for 6 h, based on our results from preliminary experiments. Those samples were used for DNA extraction.

### DNA extraction and microbiota analysis

DNA was extracted from saliva by the DNA extraction kit MORA-EXTRACT (AMR Corporation, Tokyo, Japan). The frozen DNA was mailed to the Centre for Oral Indigenous Microflora Analysis (Takamatsu, Japan). Briefly, the V3–V4 variable region of the 16S rRNA gene was amplified using primer sets 341F (NCCTACGGGAGGCAGCAG) and 806R (NGACTACHVGGGTATCTAATCC) with a polymerase chain reaction (PCR) protocol by Illumina (Illumina Inc., San Diego, CA, USA). PCR reactions were performed using KAPA HiFi Hot Start Ready mix (KAPA Biosystems Inc., Wilmington, MA, USA). PCR amplification was performed as follows: initial denaturation at 95 °C for 3 min, 28 cycles of 95 °C for 30 s, 55 °C for 30 s, 72 °C for 30 s, and a final extension step of 72 °C for 5 min. The adaptor index sequences were then assigned to identify the samples. The PCR products were purified, electrophoresed and quantified using the 1× dsDNA High Sensitivity kit via a Qubit 2.0 fluorometer (Invitrogen, Life Technologies Inc., Carlsbad, CA, USA). Each sample was adjusted to the same concentration. MiSeq (Illumina Inc., San Diego, CA, USA) was performed and each sample was corrected and mixed in equal volumes with reference to the number of reads in each. The library was denatured with 0.2 N NaOH and the concentration was adjusted to 10 pM with HT1 buffer. The obtained library was paired-end sequenced at 2 × 301 bp using a MiSeq Reagent Kit V3 (Illumina Inc., San Diego, CA, USA) and the Illumina MiSeq platform. Amplicon sequences were read and processed using UPARSE^31^. Reads of each sample were checked for quality by Fast QC scripts, and then combined with forward and reverse reads by the USEARCH and fastq_join scripts. Low-quality reads above 200 bp were removed by the QC filter script. The resultant sequences were subjected to OTU clustering to remove chimeric sequences from the data set by UCLAST with QIIME2 (ver. 1.9.1)^32^. The reads were next searched for homology by BLAST using the Greengenes1 database^33^ and the OTUs were used for analysis.

### Statistical analysis

For clinical parameters, median value was used when normality was not observed. The Mann–Whitney U test was used to compare the clinical parameters between groups. Bioinformatic analysis was conducted with QIIME2 (version 1.9.1). The species richness of each sample (alpha-diversity) was performed for the number of OTUs and by Shannon index. Kruskal–Wallis test was used for comparison between groups. Beta-diversity was performed for Principal Coordinate Analysis (PCoA) based on the OTU level with unweighted Unifrac distance. PCoA was depicted to compare each exposure group and each participant simultaneously. The statistical significance of each group was analyzed using one-way PERMANOVA test. The PERMANOVA was performed for the comparisons among the three groups (O_2_-NBW, HOCl-NBW, and Control) in the principal coordinates analysis. The repeated measures ANOVA was performed for the comparison of bacterial genus/species level abundance among the three group (O_2_-NBW, HOCl-NBW, and Control) followed by Bonferroni-Holm Correction for Multiple Comparisons for post hoc testing. Cluster analysis was performed using the Ward method according to the coordinate information calculated from PCoA (both Weighted and Unweighted). Spearman’s rank correlation was used for the correlation between the number of PD and abundance of high-susceptible bacteria*.* The significance level was set at α = 0.05. The above statistical analysis was performed using software R (ver. 4.1.2).

### Supplementary Information

Supplementary Information.

## Data Availability

The obtained sequence data were deposited in the BioProject database under accession no. PRJDB16620 in the DDBJ BioProject database.
