# Supplementary material for: The effects of exposure to O2- and HOCl-nanobubble water on human salivary microbiota
Source: Sci Rep. 2023 Nov 30;13:21125. doi: 10.1038/s41598-023-48441-6 (PMC10689733; doi:10.1038/s41598-023-48441-6)
Supplement: Supplementary file 1 — Supplementary Information. [file 41598_2023_48441_MOESM1_ESM.pdf]

Supplementary Materials

Brief title: O_2- and HOCl-nanobubble water and salivary microbiota

Figure legends for Supplementary materials

Supplementary Figure 1. Alpha-diversity of oral microbiome to exposure of NBWs.

(A) Observed features. (B) Shannon index.

Supplementary Figure 2. Relative frequencies of bacterial genera (A) and species (B). Relative abundance of oral bacterial genera (A) or species (B) in each sample for NBW exposed. Bacterial general or species with detected relative abundance ≥ 1 % were shown.

Supplementary Figure 3. Two cluster with different susceptibility to exposed O_2-NBWand HOCl-NBW in Unweighted UniFraq distance (A, B), and Weighted UniFrac distance (C, D).

(A): Control vs. O_2-NBW; CL1: $P = 0.536$, CL2: $P = 0.033$, (B): Control vs. HOCl-NBW; CL1: $P = 0.600$, CL2: $P = 0.213$. (C): Control vs. O_2-NBW; CL3: $P = 0.268$, CL4: $P = 0.283$, (D): Control vs. HOCl-NBW; CL3: $P = 0.389$, CL4: $P = 0.438$.

Supplementary Figure 4. Flow diagram of participant recruitment.

Supplementary Figure 1

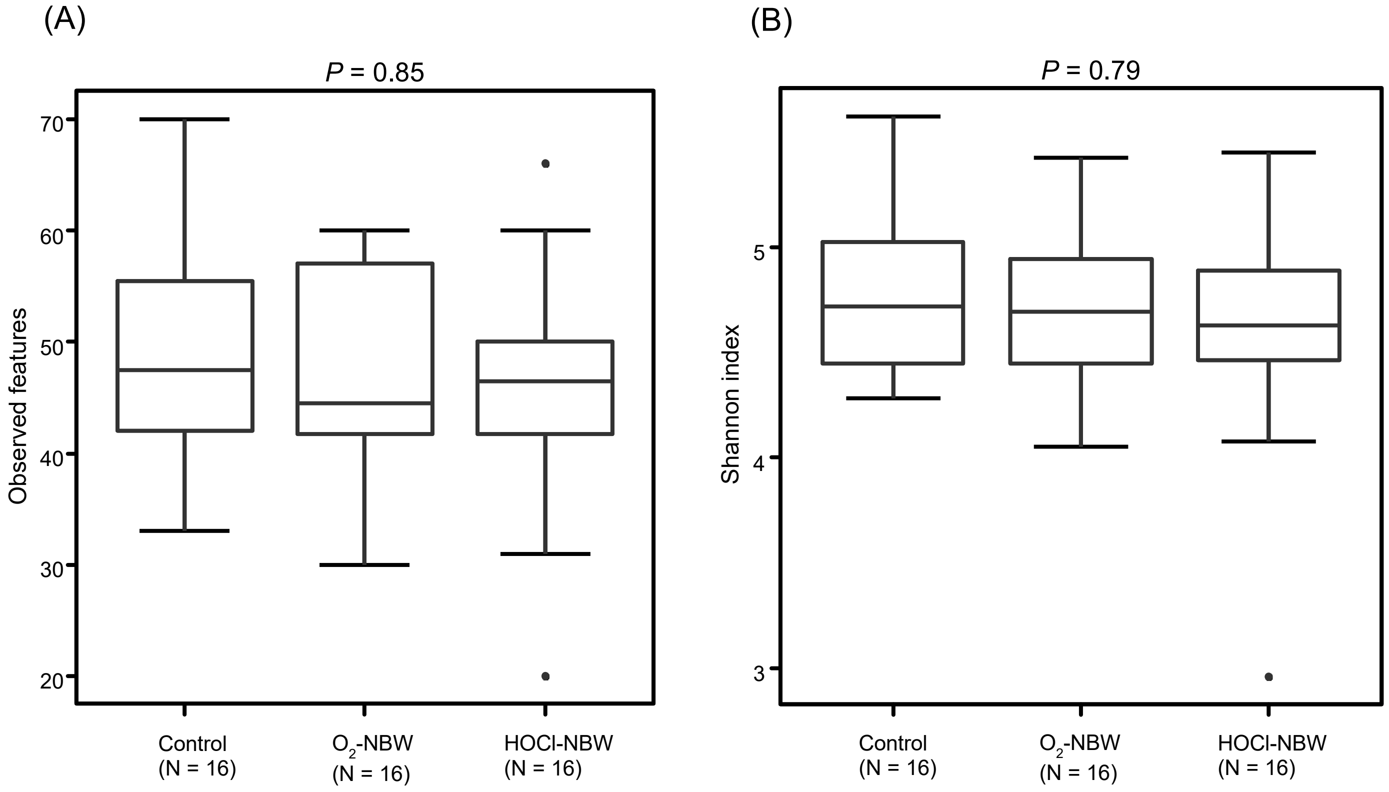

Supplementary Figure 2.

(A)

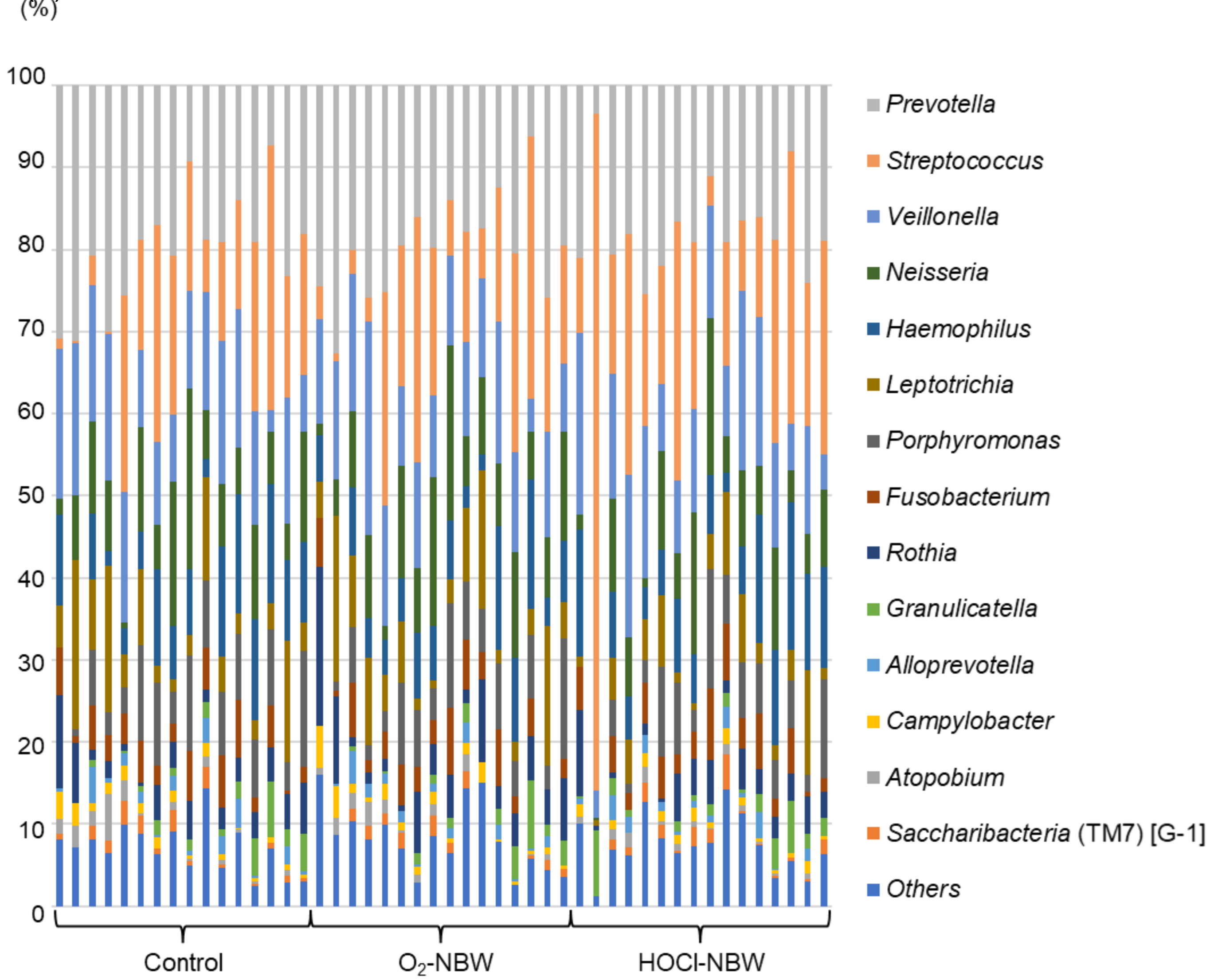

Supplementary Figure 2. (Cont'd)

(B)

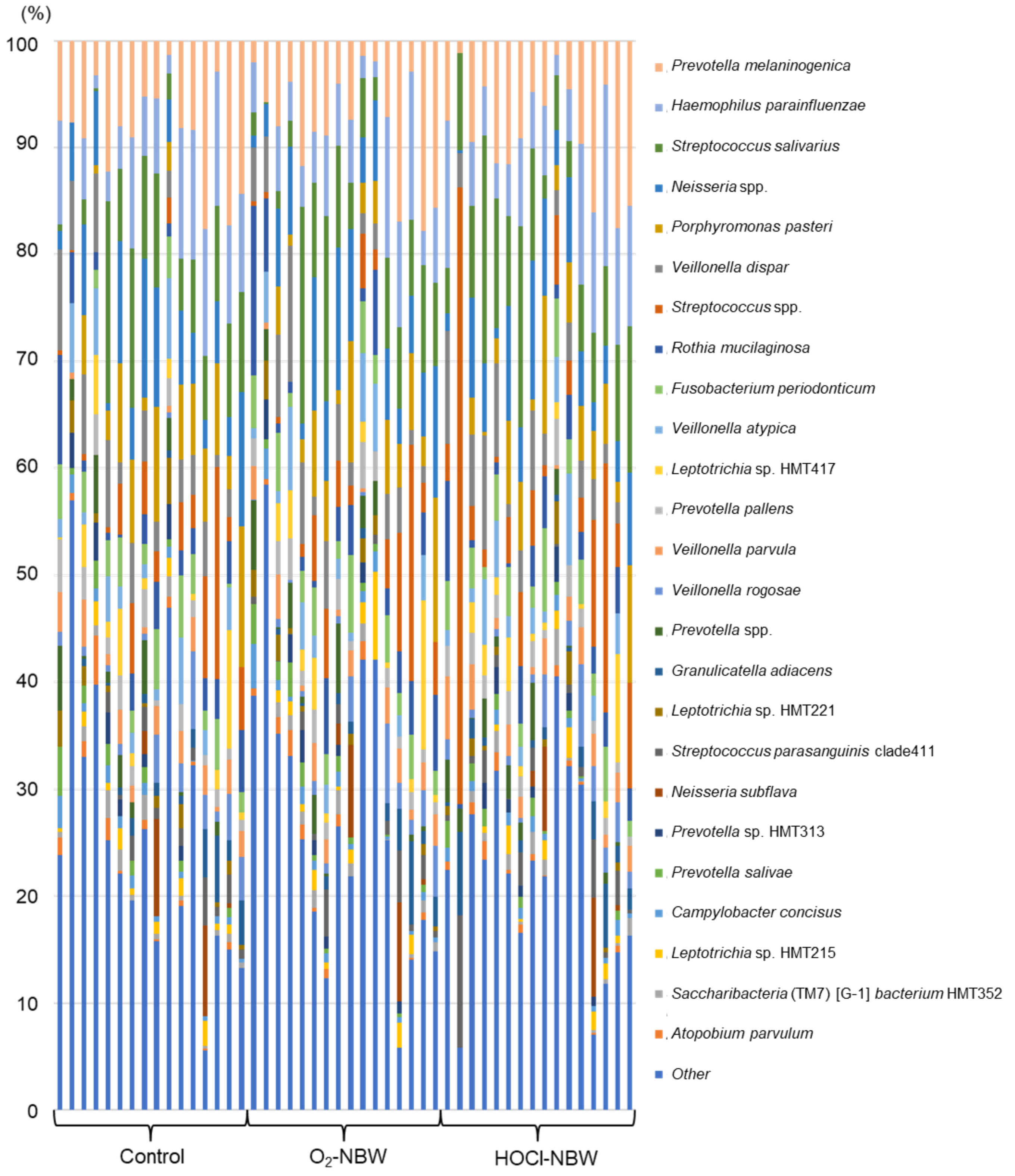

Supplementary Figure 3.

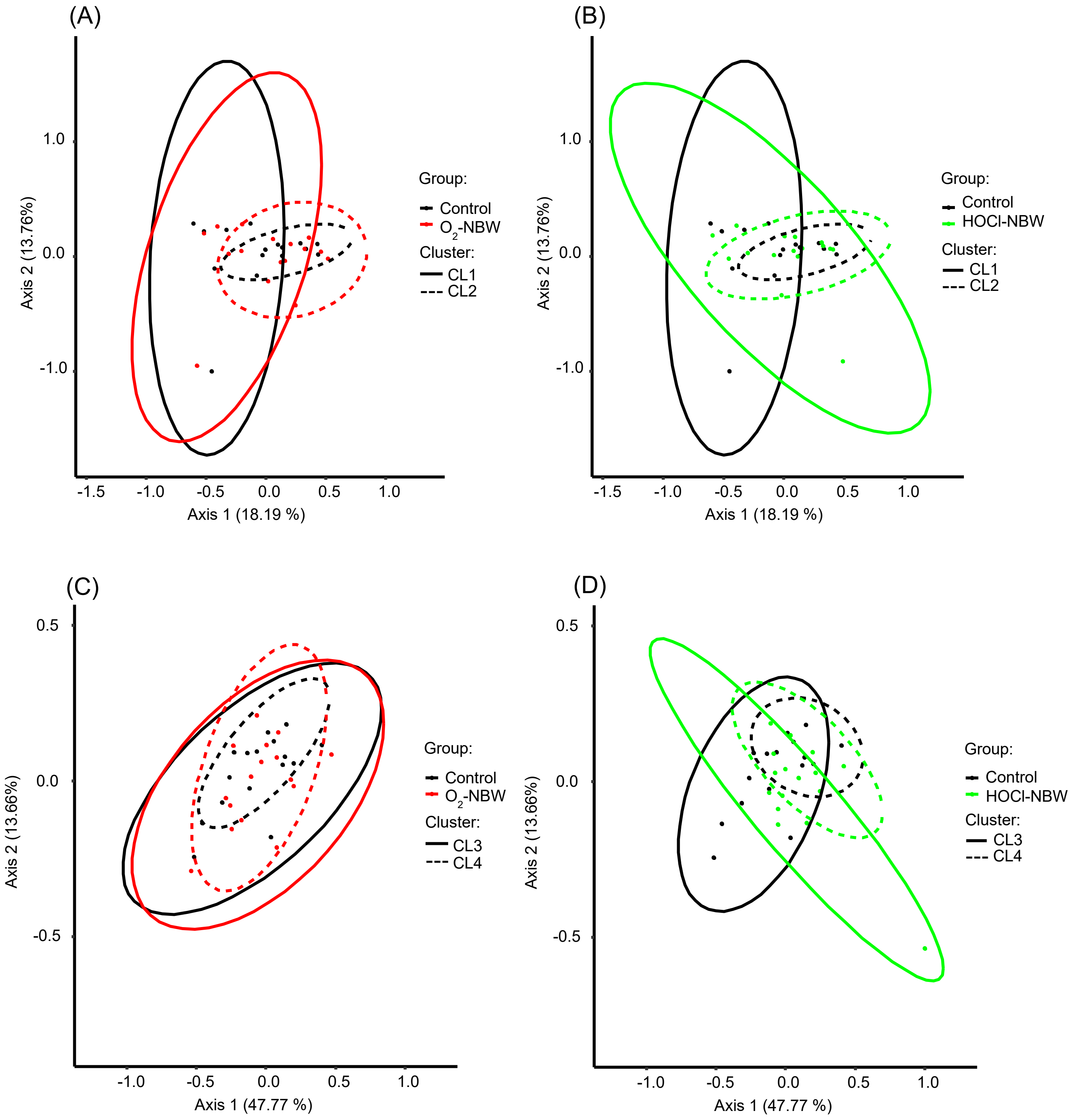

Supplementary Figure 4.

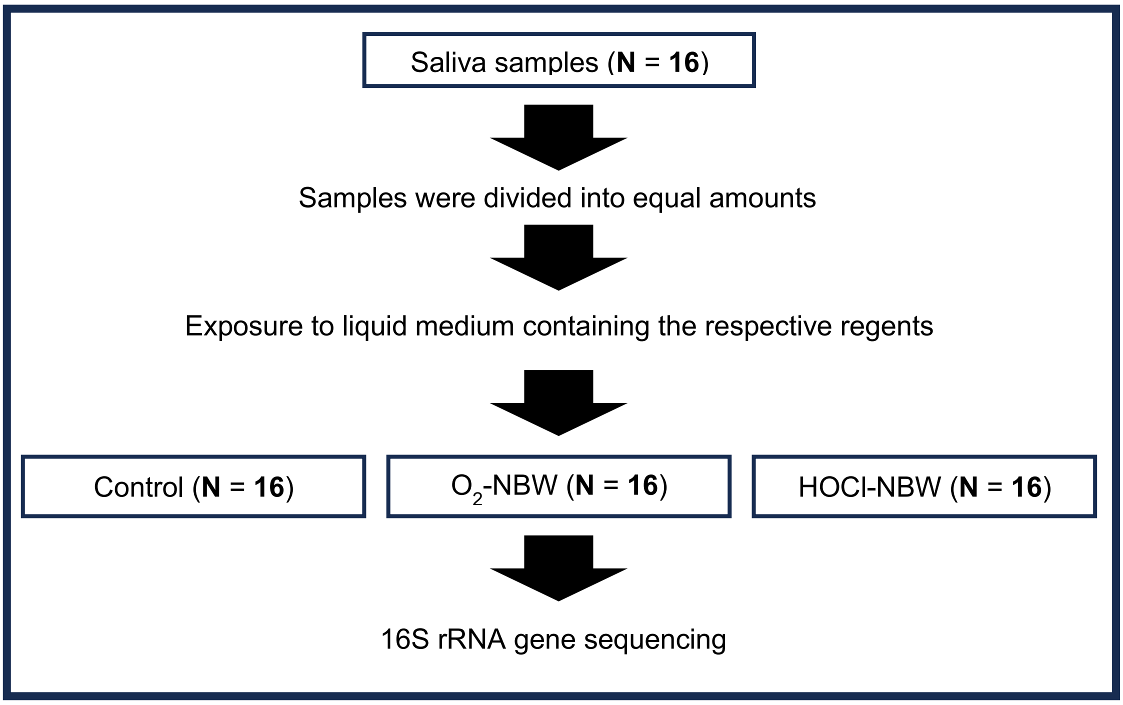

Supplementary Table 1. Relative abundance of bacterial genera and species in Unweighted UniFraq: CL1 and CL2.

(A)

Genera: CL1	Control	O_2-NBW	HOCl-NBW	O_2-NBW	HOCl-NBW
Prevotella	23.355	22.615	17.049	-	-
Streptococcus	7.206	9.252	29.349	-	-
Veillonella	14.192	14.638	11.313	-	-
Neisseria	10.656	10.335	8.777	-	-
Haemophilus	3.832	4.607	4.489	-	-
Leptotrichia	11.789	9.515	5.164	-	-
Porphyromonas	5.647	5.089	4.154	-	-
Fusobacterium	3.689	3.827	3.656	-	-
Rothia	2.579	3.176	1.270	-	-
Granulicatella	0.750	0.684	2.250	-	-
Alloprevotella	1.854	1.811	1.524	-	-
Campylobacter	1.576	1.555	1.002	-	-
Atopobium	2.101	1.787	0.939	-	-
Saccharibacteria (TM7) [G-1]	1.770	1.626	1.747	-	-

Data indicate median.
Statistical analysis was performed with repeated-measures ANOVA between control and O_2-NBW or HOCl-NBW. - : NS.

Genera: CL2	Control	O_2-NBW	HOCl-NBW	O_2-NBW	HOCl-NBW
Prevotella	18.350	18.124	17.628	-	-
Streptococcus	17.757	17.642	18.242	-	-
Veillonella	12.918	11.789	13.945	-	-
Neisseria	7.922	8.845	7.332	-	-
Haemophilus	10.837	7.937	11.066	-	-
Leptotrichia	4.426	5.882	4.250	-	-
Porphyromonas	7.444	6.483	6.330	-	-
Fusobacterium	4.099	3.992	4.169	-	-
Rothia	4.469	6.781	4.508	-	-
Granulicatella	2.426	2.143	1.939	-	-
Alloprevotella	1.127	0.646	0.972	-	-
Campylobacter	1.029	1.361	0.937	-	-
Atopobium	0.642	0.423	0.553	-	-
Saccharibacteria (TM7) [G-1]	0.731	0.538	0.749	-	-

Data indicate median.

Statistical analysis was performed with repeated-measures ANOVA between control and O_2-NBW or HOCl-NBW. - : NS.

Supplementary Table 1. (Cont'd)
(B)

Species: CL1	Control	O_2-NBW	HOCl-NBW	O_2-NBW	HOCl-NBW
Prevotella melaninogenica	7.017	6.317	6.167	-	-
Haemophilus parainfluenzae	3.596	4.364	4.332	-	-
Streptococcus salivarius	4.131	5.484	11.862	-	-
Neisseria spp.	9.856	9.523	8.204	-	-
Porphyromonas pasteri	3.839	3.436	2.891	-	-
Veillonella dispar	6.287	7.012	5.944	-	-
Streptococcus spp.	2.548	3.272	13.974	-	-
Rothia mucilaginosa	2.440	2.900	1.270	-	-
Fusobacterium periodonticum	3.211	3.541	3.348	-	-
Veillonella atypica	5.215	4.914	3.122	-	-
Leptotrichia sp. HMT417	3.715	2.910	2.488	-	-
Prevotella pallens	3.232	3.426	2.910	-	-
Veillonella parvula	1.889	1.842	1.589	-	-
Veillonella rogosae	0.597	0.654	0.452	-	-
Prevotella spp.	3.805	4.089	3.166	-	-
Granulicatella adiacens	0.588	0.510	2.104	-	-
Leptotrichia sp. HMT221	2.223	1.881	1.224	-	-
Streptococcus parasanguinis clade411	0.527	0.496	3.330	-	-
Neisseria subflava	0.400	0.386	0.269	-	-
Prevotella sp. HMT313	2.724	2.203	1.562	-	-
Prevotella salivae	1.430	1.699	1.190	-	-
Campylobacter concisus	1.452	1.555	1.002	-	-
Leptotrichia sp. HMT215	1.498	1.320	0.969	-	-
Saccharibacteria (TM7) [G-1] *bacterium* HMT352	1.728	1.626	1.747	-	-
Atopobium parvulum	1.808	1.573	0.939	-	-

Data indicate median. - : NS.

Statistical analysis was performed with repeated-measures ANOVA.

Supplementary Table 1. (Cont'd)

Species: CL2	Control	O_2-NBW	HOCl-NBW	O_2-NBW	HOCl-NBW
Prevotella melaninogenica	11.245	10.088	11.208	-	-
Haemophilus parainfluenzae	10.660	7.787	11.066	-	-
Streptococcus salivarius	9.881	9.593	9.240	-	-
Neisseria spp.	6.002	6.861	5.436	-	-
Porphyromonas pasteri	7.296	6.390	6.160	-	-
Veillonella dispar	4.715	3.943	4.596	-	-
Streptococcus spp.	6.016	6.191	7.289	-	-
Rothia mucilaginosa	4.423	6.781	4.508	-	-
Fusobacterium periodonticum	3.824	3.810	3.923	-	-
Veillonella atypica	2.340	2.467	2.754	-	-
Leptotrichia sp. HMT417	1.881	2.093	1.677	-	-
Prevotella pallens	1.946	1.854	1.908	-	-
Veillonella parvula	2.651	2.365	3.058	-	-
Veillonella rogosae	3.198	2.778	3.251	-	-
Prevotella spp.	1.142	1.415	0.915	-	-
Granulicatella adiacens	2.426	2.143	1.939	-	-
Leptotrichia sp. HMT221	1.041	0.877	0.766	-	-
Streptococcus parasanguinis clade411	1.860	1.859	1.713	-	-
Neisseria subflava	1.920	1.983	1.896	-	-
Prevotella sp. HMT313	0.477	0.580	0.698	-	-
Prevotella salivae	1.104	1.034	0.800	-	-
Campylobacter concisus	1.019	1.361	0.937	-	-
Leptotrichia sp. HMT215	0.888	1.752	1.051	-	-
Saccharibacteria (TM7) [G-1] *bacterium* HMT352	0.731	0.538	0.749	-	-
Atopobium parvulum	0.642	0.423	0.553	-	-

Data indicate median. - : NS.

Statistical analysis was performed with repeated-measures ANOVA.
